# Laboratoires médicaux et qualité des soins: la partie la plus négligée au niveau des hôpitaux ruraux de la République Démocratique du Congo

**DOI:** 10.11604/pamj.2020.35.22.18755

**Published:** 2020-01-24

**Authors:** Sylvie Linsuke, Gisèle Nabazungu, Gillon Ilombe, Steve Ahuka, Jean-Jacques Muyembe, Pascal Lutumba

**Affiliations:** 1 Institut National de Recherche Biomédicale, Kinshasa, République Démocratique du Congo; 2 Département d’Epidémiologie et Médecine Sociale, Faculté de Médicine, Université d’Anvers, Anvers, Belgique; 3 Département de Microbiologie, Faculté de Médicine, Université de Kinshasa, Kinshasa, République Démocratique du Congo; 4 Département de Médecine Tropicale, Faculté de Médicine, Université de Kinshasa, Kinshasa, République Démocratique du Congo

**Keywords:** Laboratoires cliniques, fonctionnement, qualité des soins, République Démocratique du Congo, Clinical laboratories, performance, quality of care, Democratic Republic of the Congo

## Abstract

**Introduction:**

La qualité des soins est essentielle pour sauver des vies humaines de différentes maladies. Cependant, un diagnostic inapproprié ne peut en aucun cas aboutir à une prise en charge correcte des patients ainsi qu’à des soins de qualité. Nous avons effectué une analyse descriptive transversale dans trois laboratoires des hôpitaux généraux en République Démocratique du Congo.

**Méthodes:**

Une équipe d’experts nationaux dans le domaine des laboratoires avait conduit l’enquête au niveau de trois laboratoires cliniques des hôpitaux généraux de la République Démocratique du Congo. Des observations, visites et entretiens structurés à l´aide d’un questionnaire ont été utilisées pour évaluer la performance de ces laboratoires cliniques. Nous avons également utilisé un guide d’évaluation développé au niveau national pour l’évaluation des laboratoires.

**Résultats:**

Les laboratoires cliniques des hôpitaux généraux visités ont présenté de nombreux déficits notamment en ce qui concerne les infrastructures, la formation de base et continue des personnels, les équipements, la supervision et le contrôle de qualité. Le plateau technique de ces laboratoires n’était pas adapté pour répondre aux besoins de la population en ce qui concerne les maladies fréquemment rencontrées dans ces zones. Nous avons également noté que, ces laboratoires sont peu ou presque pas accompagnés et qu’il n’y avait aucune équipe de coordination dédiée à la supervision et évaluation des laboratoires au niveau de l’hôpital, voire même au niveau de la zone de santé. En plus, les techniciens de ses différents laboratoires n’ont pas été supervisés pendant de nombreuses années.

**Conclusion:**

Les laboratoires cliniques doivent être améliorés pour permettre un diagnostic adéquat de différentes maladies. Cette amélioration doit s’appuyer sur les maladies locales. Au sein du système, il est important de consacrer plus d’attention aux laboratoires cliniques. Un plaidoyer pour cette composante négligée du système de santé est nécessaire, car cette situation pourrait être la même dans de nombreux pays en voie de développement.

## Abstract (en)

**Introduction:**

Quality of care is essential to save people living with different diseases. However, inappropriate diagnosis may in no case lead to proper patient management as well as to quality of care. We conducted a cross-sectional descriptive analysis in three laboratories at the General Hospitals in the Democratic Republic of the Congo.

**Methods:**

A team of national experts in the field of laboratories conducted a survey in the three clinical laboratories of the General Hospitals in the Democratic Republic of the Congo. Observations, visits and structured interviews using a questionnaire were used to assess the performance of these clinical laboratories. We also used a national evaluation guidance for the assessment of laboratories.

**Results:**

The clinical laboratories of the General Hospitals visited showed many deficits, in particular, in infrastructures, in the basic and continuous training of the personnel, in the equipment, in supervision and quality control. Technical performances of these laboratories were not adapted to meet the needs of the population with regard to diseases frequently encountered in these areas. We also noted that these laboratories are little or almost not assisted and that there was no coordination team dedicated to the supervision and the assessment of laboratories in the hospital or even in the health zone. In addition, technicians working in their different laboratories had not been supervised over many years.

**Conclusion:**

Clinical laboratory improvement would allow for proper diagnosis of different diseases. This improvement should take into account local diseases. Within the system, it is important to devote more attention to clinical laboratories. Advocacy for this neglected component of the health system is necessary, as this situation could be the same in many developing countries.

## Introduction

**Figure 1:**
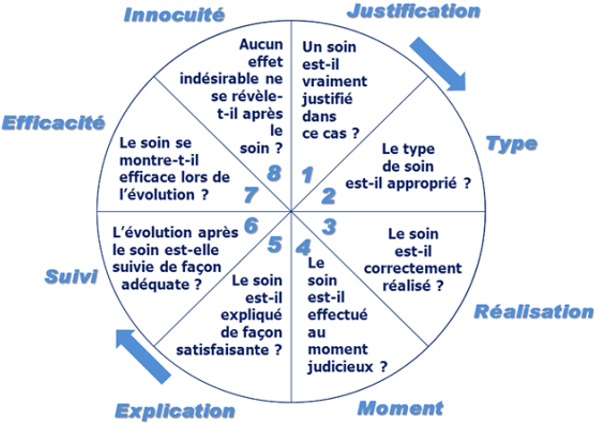
Modèle conceptuel de la qualité des soins avec ses huit composantes: modèle proposé par les hôpitaux universitaires de Strasbourg

La collaboration entre le laboratoire de diagnostic et les cliniciens est un élément essentiel pour des soins de qualité. En effet, le laboratoire permet aux cliniciens non seulement d’avoir un diagnostic précis mais aussi et surtout un bilan de santé du patient basés sur une évaluation biologique et un diagnostic précis [1]. Le laboratoire sert aussi dans la détection et confirmation des épidémies pour une bonne surveillance épidémiologique et une riposte précise et à temps [1, 2]. Pour s’y faire, les laboratoires doivent avoir un plateau technique leur permettant de jouer pleinement les trois rôles principaux à savoir le diagnostic, le bilan biologique et la surveillance épidémiologique. Outre le plateau technique, un dialogue entre le personnel de laboratoire et les cliniciens est nécessaire pour une meilleure interprétation des résultats [3]. Si dans les pays développés, la confirmation du diagnostic par des examens complé-mentaires est de mise, dans les pays sous-développés, le diagnostic est souvent présomptif et les cliniciens sont souvent obligés de traiter tous les diagnostics différen-tiels avec comme résultats des prescriptions non conformes et une mauvaise prise en charge des patients. Un exemple typique est le cas de coma chez un diabétique. En l’absence de possibilité de dosage de la glycémie, le clinicien peut se tromper facilement entre le coma hypoglycémique et hyperglycémique. Son attitude pourra ainsi aggraver le coma. Actuellement, le fonctionnement des laboratoires cliniques est sous documenté et leur apport dans la qualité de la prise en charge des patients n’est pas toujours pris à sa juste valeur dans les pays sous-développés. Plusieurs facteurs peuvent concourir à cette situation comme l’absence de laboratoires cliniques dans les milieux reculés, l’absence des équipements essentiels de base de laboratoire, des problèmes de chaîne logistique, le nombre limité de personnels qualifiés, manque ou insuffisance de la formation du personnel de laboratoire, absence ou non usage des procédures de laboratoire, défaillance de systèmes de contrôle de qualité, et autres [4-8]. Ce dysfonctionnement peut compromettre la qualité des résultats des laboratoires cliniques avec comme répercutions la perte de confiance des cliniciens mais aussi une faible qualité de la prise en charge des patients (voir le modèle conceptuel: Figure 1) [6, 8]. Le renforcement des capacités des laboratoires cliniques serait donc un maillon essentiel pour un service de santé de qualité [9]. Ceci serait donc un défi majeur à relever pour les pays sous-développés pour une meilleure prise en charge des problèmes sanitaires et de surveillance épidémiologique. Pour pouvoir améliorer les laboratoires cliniques, nous avons procédé à une évaluation initiale de laboratoires cliniques de trois zones de santé d’apprentissage (ZAR) du Projet de Renforcement Institutionnel pour des Politiques de Santé basées sur les Evidences en République Démocratique du Congo (RIPSEC) afin d’identifier leur niveau de fonctionnement dans le but de rechercher les goulots d’étranglements qui empêcheraient à ces laboratoires de jouer pleinement leur rôle dans le système de santé.

## Méthodes

**Figure 2:**
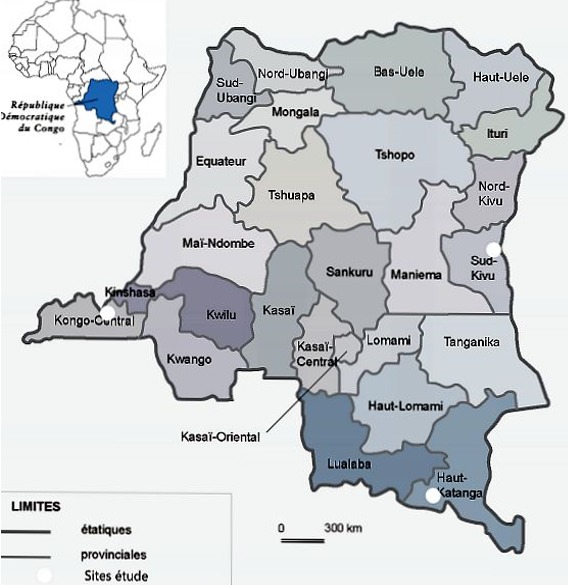
Carte de la République Démocratique du Congo indiquant les sites d’étude

Une enquête transversale descriptive a été conduite du 05 au 26 juillet 2016 respectivement, dans les provinces du Kongo-Central, Haut-Katanga et Sud-Kivu comme le montre la Figure 2. L’enquête a concerné essentiellement les laboratoires des hôpitaux généraux de référence (HGR) des trois ZAR du projet RIPSEC. Ces laboratoires ont été choisis par convenance en fonction de leur appartenance aux ZAR appuyé par le RIPSEC.

**Brève description du projet RIPSEC RDC:** RIPSEC est un projet financé par l’Union Européenne (EuropeAid/135178/C/ACT/Multi) pour contribuer au renforcent du système de santé de la République Démocratique du Congo (RDC) pour une prise en charge plus équitable de la santé de la population dans une perspective de couverture universelle santé. Le projet appui trois zones de santé de la RDC (Figure 2). Ce projet à trois objectifs spécifiques lui permettant à remplir sa mission à savoir: i) développer la capacité de gestion de connaissance en santé en RDC par la création d’un centre de connaissances santé en RDC; ii) renforcer la capacité scientifique des écoles de santé publique (ESP) et de l’institut national de recherche biomédicale (INRB) en RDC en matière de recherche et d’enseignement; iii) renforcer la capacité de formation des écoles de santé publique en RDC par la création de zones de santé de démonstration en matière de couverture universelle. Le project RIPSEC vise à renforcer les capacités institutionnelles des établissements nationaux de formation et recherche en santé publique en RDC. Ainsi, il s’appuie sur ses partenaires (des ESP de Kinshasa, Lubumbashi, Bukavu et d’INRB) pour pouvoir répondre à ses objectifs. L’INRB comme laboratoire national de référence au niveau du pays, utilise son expertise dans l’appui à la surveillance épidémiologique et technique des laboratoires cliniques de trois ZAR pour les rendre fonctionnels aussi bien pour le diagnostic clinique que pour la surveillance épidémiologique.

**Outil d’évaluation et de collecte des données:** l’enquête avait consisté en trois parties à savoir: i) interviews structurées adressées aux personnels de laboratoires; ii) observations directes des activités d’analyse biomédicale; iii) inspection du lieu de travail incluant les paillasses, les réactifs, leur stockage, les procédures standard. Une check-list de recueil d’information sur l’organisation et fonctionnement du laboratoire établi par le Réseau National de Laboratoire de Santé Publique (RNLSP) dirigée par l’Institut National de Recherche Biomédicale (INRB) a été utilisé à cette fin. Cette check-list a été élaborée conformément au guide de bonne exécution des analyses des biologies médicales (GBEA), qui spécifie les exigences de qualité et de compétence applicables aux laboratoires de biologie médicale [10-12]. La liste comportait les éléments nécessaires des systèmes d’assurance qualité et d’amélioration de la qualité au laboratoire: infrastructures, l’organisation de laboratoire et la gestion des ressources humaines, les équipements de laboratoire en état de fonctionnement, le plateau technique, les méthodes d’analyse réalisées au laboratoire, la supervision et les activités d´assurance qualité ainsi que les renseignements sur la biosécurité et sureté au laboratoire.

## Résultats

**Infrastructures de laboratoire:** les infrastructures étaient inadéquates sauf pour un seul laboratoire qui disposait d’infrastructures bien aménagées avec cinq pièces ouvertes, propres, bien aérées, éclairées et avec des zones de séparations suffisamment espacées pour les travaux de laboratoire.

**Organisation de service de laboratoire et la ressource humaine:** aucun organigramme n’était mis en place dans les trois laboratoires. Les réunions de staff ne sont pas tenues régulièrement. Le plan d’évaluation des compétences du personnel, ni celui de leur formation est inexistant avec comme conséquences qu’aucun personnel n’a bénéficié d’une formation dans les douze derniers mois précédant l’enquête. Le nombre médian était de 4 techniciens avec un minimum de 2 et un maximum de 4 (Tableau 1). Ce personnel avait des qualifications variées allant de technicien de laboratoire à biologiste médical. La responsabilité des licenciés biologistes médicaux n’était pas bien définie. Les trois laboratoires étaient tenus par des techniciens de laboratoire de niveau de graduat. Un technicien de surface (nettoyeur) était présent dans l’un des trois laboratoires enquêtés.

**Tableau 1:** Infrastructures et personnels de laboratoire

Variables	Laboratoire HGR 1	Laboratoire HGR 2	Laboratoire HGR 3
**Nombre locaux**	1	1	5
**Qualifications personnel**			
Nombre total	**2**	**4**	**4**
Technicien de laboratoire A1	2	1	3
Technicien de laboratoire A2	0	2	0
Biologiste médicale	0	1	0
Fille de salle	0	0	1
**Nombre des formations reçues une année précédant l’enquête**	0	0	0

**Equipements de laboratoire en état de fonctionnement:** les laboratoires disposaient tous d’au moins un microscope, une centrifugeuse et un agitateur. Deux laboratoires sur les trois ne possédaient pas de spectrophotomètre, distillateur, automate, incubateur et autoclave (Tableau 2). L’équipement informatique était disponible dans un seul laboratoire. La politique de gestion des équipements ainsi que de maintenance était inexistante. Les procédures opératoires standards (SOPs) pour les équipements avec la date d’achat ou d’entretien étaient retrouvées dans un laboratoire sur les trois.

**Tableau 2:** Répartition des équipements de laboratoire en état de fonctionnement au niveau des HGR visités

Equipement de laboratoire	Laboratoire HGR 1	Laboratoire HGR 2	Laboratoire HGR 3
Microscope	4	1	4
Réfrigérateur	0	1	5
Centrifugeuse	4	3	5
Rotateur ou agitateur	1	1	4
Congélateur	0	0	2
Spectrophotomètre	0	0	2
Distillateur	0	0	1
Automate	0	0	1
Incubateur	0	0	3
Incinérateur	1	0	1
Autoclave	0	0	3
Stérilisateur manuel	0	0	1
Analyseur d’urines	0	0	1
Appareil informatique	0	0	4

**Les différents types d’analyses effectuées dans le laboratoire:** le plateau technique de chaque laboratoire était fonction de la disponibilité des matériels et des ressources nécessaires disponibles à sa réalisation. Pour ces différents laboratoires, la plupart d’analyses offertes étaient des examens usuels comme le montre le Tableau 3, Tableau 3 (suite). Les examens spécialisés n’étaient disponibles que dans un seul laboratoire sur les trois laboratoires enquêtés. Les deux autres laboratoires référaient les malades dans le laboratoire d’un HGR de la zone voisine ou bien au laboratoire provincial pour des examens spécialisés.

**Tableau 3:** Plateau technique disponible au niveau des HGR

Plateau technique	Labo 1	Labo 2	Labo 3
**Hématologie**			
Dosage hémoglobine	+	+	+
Dosage hématocrite	+	+	+
Numération de globules blancs & rouges	+	+	+
Numération réticulocyte	-	-	+
Numération plaquettes	-	-	+
Formule leucocytaire	+	+	+
Vitesse de sédimentation	+	+	+
Groupage sanguin	+	+	+
Test de compatibilité	+	+	+
Temps de saignement & de coagulation	+	+	+
Test de falciformation	-	-	+
Morphologie cellulaire sur frottis sanguin	-	-	+
**Parasitologie**			
Goutte fraiche	+	+	+
Goutte épaisse	+	+	+
Frottis sanguin	+	+	+
Selles à frais	+	+	+
Selles après concentration	-	-	-
Sédiment urinaire	+	+	+
mAECT	+	-	-
Frottis vaginal	+	+	-
Spermogramme	+	+	-
**Biochimie**			
Urée	-	-	+
Créatinine	-	-	+
Glucose	-	-	+
Glycémie avec le glycomètre	-	+	-
Acide urique	-	-	+
Réserve alcaline	-	-	+
Transaminases	-	-	+
Cholestérol	-	-	+
Phosphatases alcalines	-	-	+
Bilirubine totale	-	-	+
Bilirubine directe	-	-	+
Rivalta	-	-	+
Bactériologie			
Ziehl	+	+	+
Encre de chine	-	-	+
Gram	-	-	+
Recherche de l’antigène F1 et soluble	-	-	+

**Tableau 3 (suite):** Plateau technique disponible au niveau des HGR

	Labo 1	Labo 2	Labo 3
**Sérologie**			
CATT	+	+	+
TDR paludisme	+	+	+
TDR trypanosomiase humaine africaine	+	-	-
VIH	+	+	+
Hépatites	+	+	+
Syphilis	+	+	+
Ag HBs	-	-	+
Widal	+	+	+
Test de grossesse	+	+	+
**Culture et autres**			
Isolement	-	-	+
Identification biochimique	-	-	+
Sérotypage	-	-	+
Préparation des réactifs et milieux des cultures	-	-	+
PCR pour le crachat	-	-	+

**Méthodes analytiques utilisées au laboratoire:** globalement, les méthodes utilisées par ces laboratoires étaient normalisées sauf pour certains laboratoires qui par manque d’équipements étaient obligés d’adapter certaines méthodes. En plus, certaines méthodes d’analyse ont été améliorées via des procédures d’analyses (usage des lames porte-objet par manque des lamelles couvre-objet pour couvrir les préparations de selles à frais et de Ziehl, la morphologie cellulaire a été initiée lors de la formule leucocytaire, confusion dans l’estimation de la densité parasitaire, utilisation des micropipettes disponibles pour améliorer la précision du dosage de l’hémoglobine et de la numération des globules blancs). Par ailleurs, les SOPs utilisés comme référentiels au laboratoire étaient soit existant mais non affiché ou carrément inexistant dans tous les laboratoires. Aussi, les manuels de qualité et de sécurité au laboratoire sont particulièrement absents.

**Supervision:** a part la supervision peu documentée assurée par les spécialistes des programmes spécialisés comme le VIH-SIDA, paludisme et tuberculose concernant leurs composantes, il n’a pas été rapporté des supervisions venant de la hiérarchie à savoir le bureau central de la zone de santé ou du laboratoire provincial.

**Contrôle de qualité:** les contrôles de qualité interne sont réalisés de manière partielle notamment pour les réactifs de laboratoire. Un laboratoire sur les trois réalise le contrôle de qualité de nouveaux matériels. Le contrôle de qualité externe est quasi inexistant, à l’exception de celui des tests diagnostiques rapides (TDR) (Paludisme et THA) et celui des Ziehl, organisé respectivement par les programmes spécialisés.

**Biosécurité et sureté au laboratoire:** la biosécurité était quasi inexistante dans les laboratoires enquêtés. Certains principes ou normes étaient affichés au niveau de laboratoires mais non respecté par les personnels. Les laboratoires manquent les équipements de protection individuelle.

## Discussion

A l’issue de notre étude, il a été noté que les laboratoires des HGR avaient beaucoup de déficit notamment en ce qui concerne les infrastructures, la formation de base et continue des personnels, les équipements, la supervision et le contrôle de qualité. Bien que les trois laboratoires choisis l’aient été du fait de leur appartenance aux zones d’apprentissage et de recherche du projet RIPSEC, cette situation serait probablement la même pour les autres HGR. En effet, le document du plan national de développement sanitaire de la RDC rapporte que les laboratoires des HGR manquent le plateau technique de base comprenant la parasitologie, la biochimie, la bactériologie et l’hématologie [13]. Cette situation aurait pu être atténuée si le Guide de Bonne Exécution des Analyses de biologie médicale élaboré par la direction nationale de laboratoires avait été respecté [11-13]. En plus, le financement à différents niveaux des HGR influencerait le plateau technique au niveau du laboratoire. En effet, notre étude n’a pas eu comme objectif de comparer les différents niveaux de financement et la portion attribuée aux activités de laboratoire. Cette étude serait intéressante dans le futur pour déterminer le financement nécessaire pour un laboratoire d’un HGR. Les déficits de laboratoire constatés dans notre étude ont été aussi rapportés par Bates *et al.* [4] et Petti *et al.* [6]. D’autres auteurs ont insisté sur le renforcement des laboratoires cliniques dans l’amélioration de la qualité des soins [9, 14]. Malgré la prise de conscience de l’importance des laboratoires cliniques dans l’amélioration de la qualité des soins, très peu des moyens financiers sont disponibilisés pour cela aux vues des plateaux cliniques disponibles malgré un certain appui de la zone de santé et/ou de l’HGR. Ce secteur ferait partie des secteurs négligés dont il faudrait s’occuper de manière plus spécifique en ce qui concerne le financement afin d’améliorer la prise en charge des patients. A l’heure actuelle, des efforts mondiaux sont fournis sur le plan international pour renforcer le service de laboratoire en Afrique à travers certains programmes verticaux dans la lutte contre les maladies telle que: le VIH/SIDA, la tuberculose, le paludisme et la grippe [15-17].

## Conclusion

Les différents déficits constatés au niveau des HGR compromettent la contribution des laboratoires dans la qualité des soins. En effet un mauvais diagnostic ne permet pas des meilleurs soins. Les laboratoires des HGR sont négligés parmi les autres secteurs du système de santé, malgré le rôle important qu’il joue dans la prise en charge des malades. Un appui financier supplémentaire pour ces laboratoires pourrait être important pour l’amélioration de ce secteur.

### Etat des connaissances actuelles sur le sujet

- Il est bien évident que le laboratoire médical est une composante négligée dans le système, pourtant, une composante importante dans la prise de décision thérapeutique et la surveillance épidémiologique;
- De nombreuses études menées sous d’autres cieux indiquent que les laboratoires cliniques, surtout ceux se trouvant en milieux ruraux sont moins performants pour pouvoir fournir à la population des soins de qualité;
- Il est admis actuellement que le renforcement des capacités de ces différents laboratoires est nécessaire et demeure donc un défi majeur à relever.

### Contribution de notre étude à la connaissance

- Un déficit important compromettant la qualité des soins a été constaté au niveau des laboratoires cliniques visités nécessitant ainsi une attention particulière;
- Pour garantir la qualité des soins de la population et répondre à l’objectif du millénium de la couverture universelle des soins, des investissements importants des laboratoires en termes de budget mais aussi en termes de formation, de supervision, et autres serait nécessaires.

## Remerciements

Nous remercions les membres des équipes cadres des zones de santé et les responsables de différentes structures de santé visités, les responsables de laboratoires et les mentors des ZARs de Gombe Matadi, Kisanga et Walungu pour leurs contributions dans la réussite de cette enquête. Nous remercions également la Délégation de l’Union Européenne (EuropeAid) à travers son projet de renforcement de système de santé en République Démocratique du Congo “le Projet de Renforcement Institutionnel pour des Politiques de Santé basées sur les Evidences en République Démocratique du Congo”, acronyme « RIPSEC » pour son financement.
